# Transitioning to Opioid-free Anesthesia for Pediatric Supracondylar Fracture Repairs: A Patient Safety Report

**DOI:** 10.1097/pq9.0000000000000777

**Published:** 2025-01-07

**Authors:** Laurence O. Henson, Jennifer Chiem, Emmanuella Joseph, Fiona Patrao, Daniel King-Wai Low

**Affiliations:** * From the Department of Anesthesiology and Pain Medicine, University of Washington, Seattle, Wash.; † Department of Anesthesiology and Pain Medicine, Seattle Children's Hospital, Seattle, Wash.

## Abstract

**Introduction:**

Supracondylar fractures are among the most common injuries in the pediatric population. Recently, there has been increased interest in developing opioid-free anesthetic protocols that achieve these same goals without the risks associated with opioid use, such as postoperative nausea and vomiting (PONV), delayed discharges, and respiratory depression.

**Methods:**

Seattle Children’s Hospital implemented opioid-free anesthesia (OFA) for pediatric supracondylar fracture repairs in January 2021. This patient safety report compares the clinical outcomes of these patients to those who received intraoperative opioids. Clinical effectiveness was measured using the maximum pain scores in the postanesthesia care unit (PACU), postoperative opioid rescue rates in PACU and PONV rescue rate. PACU length of stay (LOS) was chosen as a clinical balancing measure.

**Results:**

The opioid group (n = 464) had a mean maximum pain score of 3.39 compared with the OFA group (n = 816), which had a mean maximum of 3.70. The PACU IV opioid rescue rate for the opioid group was 38.82%, whereas the OFA group was 38.73%. The opioid group had a PONV rescue rate of 1.53%, compared with 0.23% in the OFA group. Mean LOS in the PACU was 79 minutes for the opioid group and 86 minutes for the OFA group.

**Conclusions:**

The shift to OFA for intraoperative management of patients’ supracondylar fracture repair resulted in similar postoperative analgesic outcomes when compared with an opioid-based approach, with a reduced PONV rate and minimal increase in LOS. Transitioning to OFA provided a safe and effective protocol for supracondylar repairs.

## INTRODUCTION

### Problem Description

Approximately 400 pediatric supracondylar fracture repair procedures are performed yearly at Seattle Children’s Hospital (SCH). Following these procedures can be challenging for pain management due to the variety of bone involvement. Traditional anesthetic management involves intraoperative opioids (typically fentanyl or morphine), with morphine being the mainstay for postprocedural pain treatment.^1^ Moreover, nonsteroidal anti‐inflammatory drugs (NSAIDS) have historically been avoided for these procedures due to concerns over bone healing.^2^ Adjuvant nonopioid analgesics have been found to greatly reduce the odds of adverse drug events for postsurgical patients.^3–6^ Across multiple studies, the use of an opioid-sparing protocol is widely effective in reducing opioid consumption for postoperative pain and opioid-related adverse drug events.^1,7,8^

### Available Knowledge and Rationale

There is an ongoing effort at SCH to transition to an OFA approach for multiple surgical procedures, including supracondylar fracture repairs.^1^ An OFA utilizing dexmedetomidine and ketorolac for tonsillectomies and adenotonsillectomies had similar mean maximum pain scores, morphine rescue rates, and postanesthesia care unit (PACU) length of stay (LOS) when compared with using intraoperative morphine.^8^ Opioid-free strabismus surgeries also have no differences in pain, opioid rescue therapy, or PACU LOS.^9^ Given that supracondylar fractures remain one of the most common procedures performed at SCH, this report sought to demonstrate that this was a safe and effective means of managing these patients.

### Specific Aim

The overall goal of this patient safety report was to demonstrate the clinical effectiveness of an OFA protocol initiated at SCH for pediatric patients’ supracondylar repairs.

## METHODS

### Context

SCH main campus is in Seattle, Wash. SCH receives complex pediatric surgical patients or surgeries throughout the Pacific Northwest, including those in the Washington, Wyoming, Alaska, Montana, and Idaho states. Anesthesiologists and certified nurse anesthetists (CRNAs) meet regularly to discuss best practices and have developed standardized clinical protocols (SCPs) for high‐volume surgeries to optimize delivery and consistency of patient care. Because SCH clinicians embrace SCPs and can track adoption and clinical effectiveness, they can develop, deploy, measure, and rapidly evaluate new SCPs.^10^

As part of the cumulative efforts of SCH to reduce opioid usage throughout our system, we adopted an OFA protocol for pediatric patients undergoing percutaneous pinning of supracondylar fractures. This report describes the clinical outcomes following the adoption of this OFA protocol. From January 1, 2018, to October 30, 2023, all patients who underwent closed reduction percutaneous pinning of a supracondylar repair were included in this safety report. On January 1, 2021, there was a shift in clinical practice at SCH to move away from intraoperative opioids toward intraoperative OFA.^6–9^

### Intervention

Before January 1, 2021, the standard anesthesia protocol for supracondylar fracture repairs involved the intraoperative administration of 0.1 mg/kg intravenous (IV) morphine or 1 µg/kg fentanyl and 15 mg/kg IV acetaminophen. After discussion with the SCH anesthesiology group, CRNAs, and perioperative nurses, we changed the protocol to preoperative 10 mg/kg oral ibuprofen and intraoperative IV dexmedetomidine 1 µg/kg. Routine intraoperative administration of ondansetron (0.15 mg/kg) and IV fluids (10–20 mL/kg) was unchanged. Given SCH’s prior success with implementing similar OFA protocols for other common procedures, such as tonsillectomies and strabismus repairs, there was good buy-in from the rest of the perioperative team.^8,9^ There were also no issues with surgeon buy‐in for this new protocol, thanks to the collaborative relationship between orthopedic surgeons and anesthesiologists at SCH.

OFA was defined as any surgery where no intraoperative opioids (fentanyl, morphine, alfentanil, hydromorphone) were used. The intraoperative nonopioid analgesic alternatives included a combination of dexmedetomidine (0.5–1 µg/kg), acetaminophen (12.5–15 mg/kg), and ketamine (0.5 mg/kg)—exact doses were left to the clinical judgment of the anesthesiologist. Intraoperative ketorolac (0.5 mg/kg) was also available if oral ibuprofen was not administered preoperatively.

### Clinical Effectiveness Measures

To evaluate the analgesic effectiveness of the OFA protocol, we used:

1. Maximum pain score in a PACU was the highest pain score recorded by a PACU nurse during the patient’s PACU stay. The maximum pain score in the PACU was selected as a primary outcome measure to assess the efficacy of the analgesic protocols. PACU nurses recorded pain scores using the faces, legs, activity, cry, consolability tool, faces pain scale-revised, or a numerical 0–10 visual analog scale.^11,12^ Each was converted to a 0- to 10-point scoring system for the analyses.
2. Postoperative IV opioid rescue rate in PACU was a dichotomous (yes/no) measure indicating if the patient received any IV opioid (fentanyl, morphine, and hydromorphone) during their PACU stay for treatment of moderate-to-severe pain.
3. Postoperative nausea and vomiting (PONV) rescue rate was a dichotomous (yes/no) measure if the patient received any antiemetic (ondansetron, metoclopramide, and diphenhydramine) during their PACU stay.

### Clinical Balancing Measures

1. PACU length of stay (LOS) was defined as the total time spent in PACU (from admit time to discharge time in minutes). This measure was chosen as it reflected both clinical effectiveness and safety and the unit’s operational efficiency (eg, if delirium rates increased, PACU LOS would be expected to increase).

### Analyses

The perioperative electronic medical record Epic (Epic Systems, Inc., Verona, Wis.) at Seattle Children’s Surgery Center flows into the hospital’s enterprise data warehouse. AdaptX (Seattle, Wash.) is a software solution that allows clinicians to use aggregated, de-identified health information from the enterprise data warehouse to understand the comparative effectiveness of variants in practice. With AdaptX, we can use real-world data to monitor the effects of protocol changes in near real-time using statistical process control charts.^13^

Mean maximum pain scores in the PACU and mean postoperative opioid rescue rates in the PACU were then presented at quarterly intervals. Given the nature of this data (nonrandomized real-world data with no exclusion criteria), we chose to present the data as statistical process control charts. We used X-bar charts for continuous data (PACU LOS, maximum pain score in PACU). P-charts were used for dichotomous data (PONV rate, IV opioid rescue rate in PACU); control limits were set at 3 sigma in both instances.

## RESULTS

**Fig. 1.**
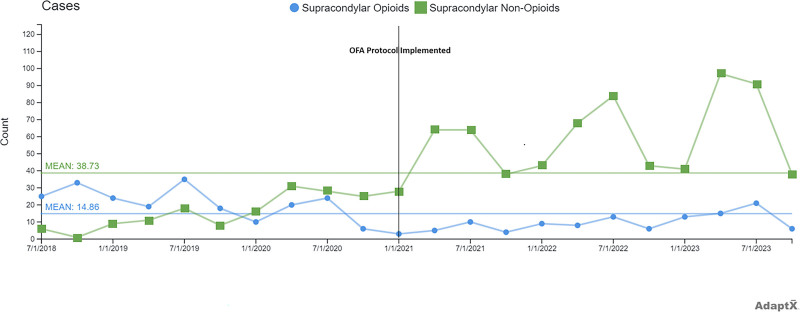
A C-chart showing the trend in cases utilizing opioid versus OFA over time.

There were 464 patients in the opioid protocol and 816 patients in the opioid-free anesthesia (OFA) protocol. Demographic data for each cohort can be seen in Table 1. Figure 1 is a C-chart showing quarterly case counts of patients undergoing supracondylar percutaneous pinning stratified by those who received opioids versus those who received OFA. We have annotated when the OFA protocol was formally implemented at SCH for the reader’s convenience.

**Table 1.** Patient Demographics, January 1, 2018–October 30, 2023 (n = 1,280)

Characteristic	Supracondylar Opioids (N = 464)	Supracondylar Nonopioids (N = 816)
Demographics		
Age groups		
Infant (0 y)	3 (0.65%)	4 (0.49%)
Toddler (1–3 y)	58 (12.5%)	148 (18.14%)
Preschool (4–5 y)	127 (27.37%)	219 (26.84%)
Childhood (6–12 y)	252 (54.31%)	412 (50.49%)
Adolescence (13–18 y)	24 (5.17)	32 (3.92%)
Other	0	1 (0.12%)
Patient birth sex		
Male	236 (50.86%)	446 (54.66%)
Female	227 (48.92%)	370 (45.34%)
Choose not to disclose	1 (0.22%)	0
Patient race and ethnicity		
Non-Hispanic White	236 (50.86%)	389 (47.67%)
Hispanic	62 (13.36%)	112 (13.73%)
Asian	56 (12.07%)	111 (13.6%)
Unknown/refused	37 (7.97%)	87 (10.66)
2 or more races	30 (5.47%)	45 (5.51%)
Other	43 (9.27%)	72 (8.82%)
BMI groups, kg/m^2^		
0–18.4	299 (64.44%)	659 (80.76%)
18.5–24.9	63 (13.58%)	82 (10.05%)
25–29.9	11 (2.37%)	13 (1.59%)
30–34.9	4 (0.86%)	5 (0.61%)
40–125	3 (0.65%)	2 (0.25%)
Other	2 (0.43%)	1 (0.12%)
ASA score		
1	282 (60.78%)	547 (67.03%)
1E	91 (19.61%)	119 (14.58%)
2	70 (15.09%)	121 (14.83%)
2E	12 (2.59%)	14 (1.72%)
3	6 (1.29%)	12 (1.47%)
Other	3 (0.65%)	3 (0.37%)
Local infiltration used		
Yes	1 (0.2%)	11 (1.3%)
No	463 (99.8%)	805 (98.7%)

ASA, American Society of Anesthesiologists.

**Fig. 2.**
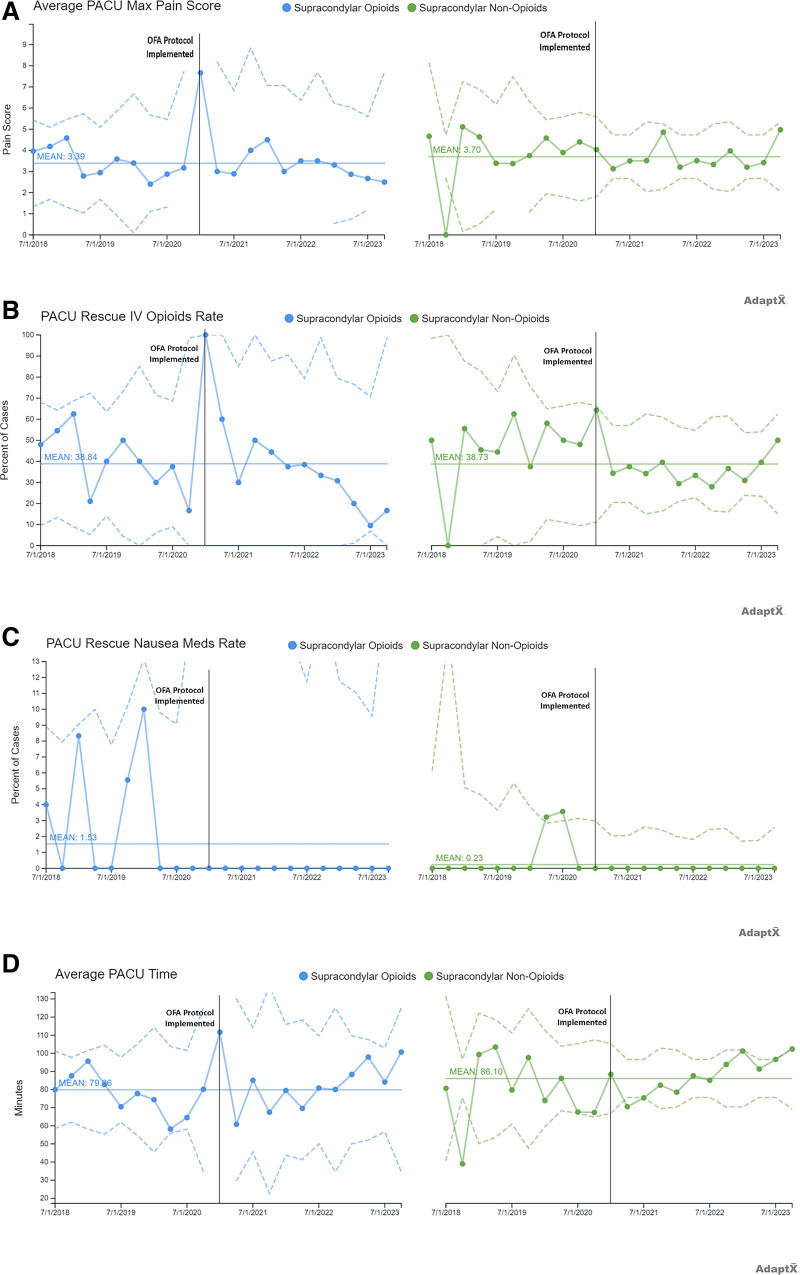
Primary and secondary outcome measures. A, An X-bar chart showing the maximum pain score in the PACU when stratified by whether the patient received opioids (mean 3.39) or OFA (mean 3.70). B, A P-chart showing the average IV opioid rescue rate when stratified by whether the patient received opioids (mean 38.34%) or OFA (mean 38.73%). C, A P-chart showing the PACU PONV rescue rate when stratified by whether the patient received opioids (mean 1.53%) or OFA (mean 0.23%). D, An X-bar chart showing the mean PACU LOS when stratified by whether the patient received opioids (79.86 min) or OFA (86.10 min).

Figure 2A–C shows the report’s clinical effectiveness measures. The maximum pain score in the PACU, when stratified by whether the patient received opioids, had a mean of 3.39, whereas the OFA group had a mean of 3.70. The average IV opioid rescue rate, when stratified by patients who received opioids, had a mean of 38.34%, and those who received OFA had a mean of 38.73%. The PACU PONV rescue rate, when stratified by patients who received opioids, had a mean of 1.53%, and those who received OFA had a mean of 0.23%.

Figure 2D shows our clinical balancing measure, with an x-bar chart demonstrating the mean PACU LOS stratified by patients who received opioids (79.86 min) and those who received OFA (86.10 min).

## DISCUSSION

### Summary

The shift in practice to OFA for patients undergoing percutaneous pinning of supracondylar fractures has been successful from both a clinical effectiveness and patient experience perspective. The data demonstrate no clinically meaningful differences in pain scores or IV opioid rescue rate but show a markedly improved PONV rescue rate. We achieved this result with a negligible difference in PACU LOS.

### Interpretation

Two of our outcome measures were designed to capture the clinical analgesic effectiveness of the OFA protocol. As shown in Figure 2A, the data stratified by patients receiving opioids versus OFA, there was no special cause variation *between* these 2 groups. This deeper interpretation of the data was made possible by extending the mean of the opioid group and noting the absence of special cause variation in the OFA group relative to this extended mean.

At SCH, the PONV rate for these patients (1.53%) was already low before adopting the OFA protocol. There were multiple factors associated with PONV, such as volatile anesthetic use, opioids, pain, ketamine, and excessive positive pressure with bag-mask ventilation.^8,9^ It was not surprising to see PONV rates decrease with the elimination of opioids. However, the magnitude of the change (almost 7-fold lower at 0.23%) with the switch to OFA was very notable. We posit that this reduction was due to the pharmacological effect of dexmedetomidine—a drug known to have analgesic properties and an independent antiemetic effect.^14^ Dexmedetomidine is also known to minimize the alveolar concentration of anesthetics, which may have reduced the amount of volatile gas needed.^15^ Outside of these changes, we made no other changes to the standard intraoperative anesthetic protocol, which included the administration of ondansetron and IV fluids.

In our current healthcare system, we must strive to improve our clinical outcomes without causing detriment to our operational efficiency. In Figure 2D, we can see that in our chosen balancing measure of PACU LOS, there was no clinically meaningful difference between the 2 groups (79.86 versus 86.1 min). In addition, a deeper analysis by extending the mean of the opioid group revealed that there was no special cause variation between those patients who received intraoperative opioids versus OFA. We note that over the past year, there has been a gradual increase in the LOS in both groups of patients, as seen in Figure 2D, most likely explained by the opening of a new operating suite with new staff and equipment at SCH in December 2020. This impact can also be seen in the sudden shift in PACU PONV rescue rate, likely due to the new suites requiring new staff training.

### Limitations

This project has several limitations because of its use of real-world data (defined as data that was routinely collected as part of receiving care in a modern digitized healthcare system). As such, there was no attempt to adjust for confounders such as emergence delirium, procedure/anesthesia duration, transportation time, and discharge readiness time, all of which may prolong PACU LOS. Another limitation was the method by which we implemented the OFA protocol. This process was a gradual shift in practice; it was not mandated that staff adopt the protocol, instead relying on socialization and diffusion of the concept through our team—consequently, the OFA protocol was not universally adopted by all staff. Additional challenges include constant staff turnover—new attendings, CRNAs, fellows and residents all introduce clinical variability into the system. Furthermore, in examining the data, we know there is variability within the OFA group (eg, dosing of dexmedetomidine, use of ketamine as an adjunct, and total intravenous anesthesia). Despite these limitations, as of the end of 2023, more than 90% of patients undergoing surgical repair of supracondylar fractures at SCH now have OFA.

### Conclusions

We created a safe and effective OFA protocol for supracondylar percutaneous pinning. Our next step is to quantify the interclinician variability that currently exists for drug selection and dosing. Once we have identified the clinicians with the best outcomes (positive deviants) in our system, we can use their practice to further improve our OFA protocol to deliver the best possible patient outcomes.

## References

[1] Martin LD Franz AM Rampersad SE. Outcomes for 41 260 pediatric surgical patients with opioid-free anesthesia: One center’s experience. Paediatr Anaesth. 2023;33:699–709. 37300350 10.1111/pan.14705

[2] Johnson MA Andras LM Andras LE. What’s new in pain management for pediatric orthopaedic surgery. J Pediatr Orthop. 2021;41:e923–e928. 34469397 10.1097/BPO.0000000000001956

[3] Imani F Zaman B De Negri P. Postoperative pain management: role of dexmedetomidine as an adjuvant. Anesth Pain Med. 2020;10:e112176. 34150582 10.5812/aapm.112176 PMC8207883

[4] Low YH Gan TJ. NMDA receptor antagonists, gabapentinoids, α-2 agonists, and dexamethasone and other non-opioid adjuvants: do they have a role in plastic surgery? Plast Reconstr Surg. 2014;134(4 Suppl 2):69S–82S. 25255009 10.1097/PRS.0000000000000703

[5] Vaughns JD Martin C Nelson J. Dexmedetomidine as an adjuvant for perioperative pain management in adolescents undergoing bariatric surgery: An observational cohort study. J Pediatr Surg. 2017;52:1787–1790. 28465076 10.1016/j.jpedsurg.2017.04.007

[6] Voepel-Lewis T Wagner D Burke C. Early adjuvant use of non-opioids associated with reduced odds of serious postoperative opioid adverse events and need for rescue in children. Paediatr Anaesth. 2013;23:162–169. 22978850 10.1111/pan.12026

[7] Peng K Ji FH Liu HY. Effects of perioperative dexmedetomidine on postoperative mortality and morbidity: a systematic review and meta-analysis. Clin Ther. 2019;41:138–154.e4. 30528108 10.1016/j.clinthera.2018.10.022

[8] Franz AM Dahl JP Huang H. The development of an opioid-sparing anesthesia protocol for pediatric ambulatory tonsillectomy and adenotonsillectomy surgery—a quality improvement project. Paediatr Anaesth. 2019;29:682–689. 31077491 10.1111/pan.13662

[9] Chiem JL Donohue LD Martin LD. An opioid-free anesthesia protocol for pediatric strabismus surgery: a quality improvement project. Pediatr Qual Saf. 2021;6:e462. 34476314 10.1097/pq9.0000000000000462 PMC8389911

[10] Langley GJ Moen RD Nolan KM. The Improvement Guide: A Practical Approach to Enhancing Organizational Performance. 2nd edn. Jossey‐Bass; 2009.

[11] Merkel SI Voepel‐Lewis T Shayevitz JR. The FLACC: a behavioral scale for scoring postoperative pain in young children. Pediatr Nurs. 1997;23:293–297. 9220806

[12] Cohen LL Lemanek K Blount RL. Evidence‐based assessment of pediatric pain. J Pediatr Psychol. 2008;33:939–955; discussion 956. 18024983 10.1093/jpepsy/jsm103 PMC2639489

[13] Liu F Panagiotakos D. Real-world data: a brief review of the methods, applications, challenges and opportunities. BMC Med Res Methodol. 2022;22:287. 36335315 10.1186/s12874-022-01768-6 PMC9636688

[14] Patel B, . Evidence Based Outcomes Center (EBOC) in collaboration with Texas Children’s Hospital. Procedural Sedation Evidence-based Guideline. Texas Children’s Hospital. 2023. Available at https://www.texaschildrens.org/sites/default/files/uploads/documents/outcomes/standards/Procedural%20Sedation%20Guideline%20FINAL%202023.pdf. Accessed August 7, 2024.

[15] Dalaei D Modir H Pazoki S. The therapeutic antiemetic and hemodynamic effects of dexmedetomidine, ephedrine, and dexamethasone in combination with midazolam on laparoscopic cholecystectomy patients: a randomised clinical trial. J West Afr Coll Surg. 2022;12:96–103. 10.4103/jwas.jwas_133_22 PMC9536412 36213814

